# Centrifugal Fiber-Spinning Device Using Two Pairs of Counter-Facing Syringes for Fabricating Composite Micro/Nanofibers and Three-Dimensional Cell Culture

**DOI:** 10.3390/polym18010016

**Published:** 2025-12-21

**Authors:** Asuka Shinagawa, Shogo Miyata

**Affiliations:** 1 Graduate School of Science and Technology, Keio University, 3-14-1 Hiyoshi, Kohoku-ku, Yokohama 223-8522, Kanagawa, Japan; 2 Faculty of Science and Technology, Keio University, 3-14-1 Hiyoshi, Kohoku-ku, Yokohama 223-8522, Kanagawa, Japan

**Keywords:** centrifugal force, fibrous scaffold, composite micro/nanofiber, tissue engineering, myoblast

## Abstract

Biomimetic scaffolds are required in tissue engineering to provide structural support as well as promote cell adhesion, proliferation, and differentiation. Fibrous scaffolds composed of micro- and nanofibers replicate the architecture of the native extracellular matrix. Electrospinning is widely used for fabricating nanofibers; however, constructing fibrous scaffolds that integrate multiple fiber scales into a single structure is difficult. We addressed this issue by developing a fiber-spinning device using two pairs of counter-facing syringes that simultaneously produce micro- and nanofibers under different processing conditions. Poly(ε-caprolactone) solutions are ejected through needle-type nozzles via centrifugal force, and fiber diameter is controlled by adjusting the polymer concentration and nozzle diameter. We fabricated scaffolds with the proposed device, which exhibited a random three-dimensional fibrous network in which microfibers and nanofibers were homogeneously integrated. C2C12 myoblasts cultured on the composite scaffolds strongly adhered to the fibrous network, remained viable, and extended along the fibers to form multinucleated cells within the structure. The developed system produced composite micro/nanofiber scaffolds with tunable morphology and biocompatibility, providing a platform for fibrous tissue engineering applications.

## 1. Introduction

Tissue engineering is a therapeutic approach that aims to regenerate or replace damaged tissues and organs through the integration of living cells, scaffolds, growth factors, and physical stimuli. The scaffold plays a vital role by providing a three-dimensional environment that supports cell adhesion, proliferation, and differentiation while mimicking the structure and functions of the native extracellular matrix (ECM). Tissue engineering commonly reconstructs tissues using combinations of cells, scaffold materials, and cytokines, where cell adhesion to scaffold materials promotes matrix synthesis and tissue remodeling.

A wide range of biodegradable polymers has been used as scaffold materials, broadly classified into natural and synthetic polymers. Natural polymers such as collagen, gelatin, and polypeptides are often processed into hydrogels or porous structures owing to their intrinsic biocompatibility and bioactivity [1,2,3]. In contrast, synthetic biodegradable polymers—including poly(L-lactic acid) (PLLA), polycaprolactone (PCL), polyglycolic acid (PGA), and poly(lactic-co-glycolic acid) (PLGA) [4,5,6,7]—offer greater tunability in mechanical properties, degradation rates, and processing versatility for fabricating well-defined scaffold architectures. Among these scaffold types, fibrous scaffolds have attracted considerable attention due to their close structural resemblance to the native ECM. Various forms of fibrous scaffolds have been reported, including non-woven mats [8], random or aligned nanofiber sheets [9], microfiber meshes [10], multilayered electrospun constructs [11], and bundled fibers designed to mimic tendon- or nerve-like tissues [12]. These fibrous scaffolds offer tunable porosity suitable for regulating cell migration, nutrient transport, and 3D tissue organization [13,14].

Electrospinning has been widely used to fabricate nanofibrous scaffolds owing to its ability to produce highly fine and uniform fiber networks, making it particularly suitable for forming thin fibrous membranes [15,16,17,18]. To overcome the relatively low throughput of conventional electrospinning, several high-productivity systems—such as multi-needle [19] and needleless [20] configurations—have been developed. Furthermore, methods for creating thicker electrospun constructs have been reported [21], including multilayer stacking [11,22]. Attempts to incorporate multiple fiber scales have also been made; for example, multilayered micro- and nanofiber constructs have been fabricated by alternately depositing microfiber and nanofiber layers, thereby combining the advantages of both length scales [22]. Nanofibers can replicate the fine fibrillar structure of collagen or other fibrous ECM and provide a large surface area for cell–material interactions, whereas microfibers contribute to mechanical stability, pore formation, and improved cellular infiltration.

Despite these advancements, electrospinning still faces inherent limitations when the goal is to generate microfibers and nanofibers simultaneously and to integrate them homogeneously within a single scaffold. Electrospinning inherently favors the formation of continuous fibers with uniform diameters under fixed electric field and solution conditions, making the concurrent formation of multiple fiber scales technically challenging. Therefore, a fabrication strategy capable of producing composite micro/nanofiber scaffolds in a single step to enable uniform spatial distribution of both fiber types is required.

Forcespinning is a centrifugal fiber fabrication technique developed as an alternative to conventional electrospinning systems [23,24]. Unlike electrospinning—which relies on electrostatic attraction between a charged polymer jet and an oppositely charged collector—forcespinning generates fibers by applying centrifugal forces to a polymer solution or melt. In this method, a spinneret loaded with the polymer fluid is rotated at high speed, and the material is extruded through a needle-type nozzle by the outward radial force. The extruded jet is stretched and thinned during its flight, after which the solvent rapidly evaporates (or the melt solidifies), allowing continuous fibers to deposit onto a surrounding collector.

Because forcespinning does not depend on high-voltage electric fields, it eliminates several limitations inherent to electrospinning, such as the requirement for dielectric solvents and restricted processability of polymer melts. Process parameters that strongly influence fiber formation include the applied centrifugal force (rotational speed), solution viscosity, solvent evaporation rate, and nozzle radius. Forcespinning also offers advantages in forming thick three-dimensional fibrous structures, as fibers are deposited in a highly porous volumetric manner rather than as thin two-dimensional membranes [25]. However, despite its potential, forcespinning has not yet been applied to produce hybrid micro/nanofiber composites to enable simultaneous and homogeneous integration of different fiber diameter scales within a single scaffold.

In this study, we developed a centrifugal-force-based fiber fabrication device that produces fibrous scaffolds containing micro- and nanofibers within a single scaffold. The morphology of the fibers produced with this device can be precisely controlled by modulating the properties of the polymer solution and parameters of the device. Cell-culture scaffolds were fabricated with the developed device, and their physical properties such as porosity and contact angle were evaluated. We further validated the biological performance of these scaffolds using C2C12 myoblasts: the cells adhered and proliferated, and myotubes formed within the fibrous matrix.

## 2. Materials and Methods

### 2.1. Materials

We used poly(ε-caprolactone) (PCL; molecular weight (Mw) = 80,000 g/mol; Sigma-Aldrich, St. Louis, MO, USA) as the polymer base and 2,2,2-trifluoroethanol (Nacalai-Tesque, Kyoto, Japan) as the solvent. Polymer solutions were prepared at concentrations of 9.5, 11, and 12 wt. %.

### 2.2. Fiber-Spinning Device Using Two Pairs of Counter-Facing Syringes for Simultaneously Fabricating Micro- and Nanofibers

**Figure 1:**
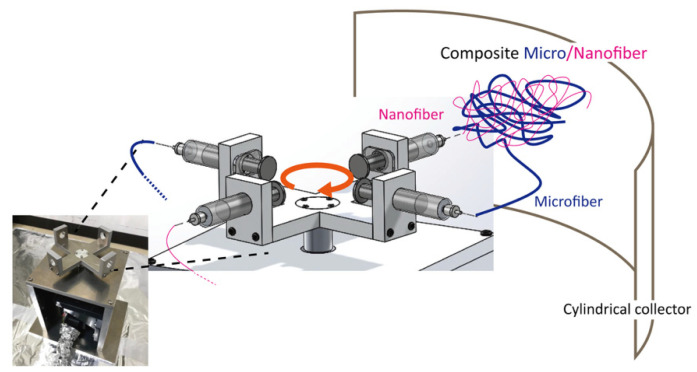
Schematic of fiber-spinning device using two pairs of counter-facing syringes for fabricating micro- and nanofibers.

A custom centrifugal-force-based fiber-spinning apparatus was developed for simultaneously fabricating fibers with different diameters. The spinneret housing was designed to mount two pairs of opposing 5 mL syringes (TERUMO, Tokyo, Japan), each equipped with a replaceable stainless-steel needle with a selectable inner diameter (22G: 0.55 mm, 21G: 0.70 mm, or 20G: 0.90 mm). A schematic of the device and its multiple-spinning mechanism is shown in Figure 1. The system enables dual spinning as each pair of syringes can be independently loaded with different concentrations of polymer solutions and nozzle sizes. Microfibers and nanofibers are concurrently generated within a single operation. The rotational speed was increased from 0 to 3000 rpm over a controlled acceleration period of 6 s, after which the system was maintained at 3000 rpm for fiber fabrication. Polymer solutions were continuously ejected from the needle-type nozzles for 300 s, during which centrifugal forces stretched the polymer jets to form fibers before deposition onto the surrounding collector. The fibers were collected on an aluminum cylindrical collector positioned 120 mm from the spinneret. The fabrication of fibers was performed at a room temperature of 24–25 °C and a relative humidity of 45–55%.

### 2.3. Fabricating and Characterizing Single-Mode Micro- and Nanofibers

PCL solutions (9.5, 11, or 12 wt.%) were individually loaded into a syringe and spun at various nozzle diameters (0.55, 0.70, and 0.90 mm) to produce single-mode fibers. The fiber morphology and diameter were evaluated under different processing conditions to establish the baseline fabrication parameters.

The fiber morphology was observed using a scanning electron microscope (FEI INSPECT S50, Thermo Fisher Scientific, Waltham, MA, USA) operated at an accelerating voltage of 10 kV. Prior to imaging, samples were coated with a thin osmium layer using an osmium coater. Osmium coating was selected because it provides uniform deposition on complex three-dimensional fiber networks and minimizes thermal damage, which is essential when imaging polymeric or biological specimens with delicate micro/nanostructures. This conductive coating enables high-resolution imaging while preserving the intrinsic morphology of the fibers. The fiber diameters were quantified from at least 100 randomly selected fibers per sample using ImageJ software v. 1.52 (NIH, Bethesda, MD, USA).

The porosity of the scaffolds was determined using the liquid displacement method, which involved immersing a porous sample in a wetting liquid, such as ethanol, to fill all void spaces within the structure. The samples were weighed before and after immersion, and the volume of the displaced liquid was considered the total pore volume. The porosity (*ε*) is calculated as the ratio of the pore volume to total volume of scaffold using the following equation:

(1) $$\varepsilon =V_{EtOH}/\left(V_{EtOH}+V_{PCL}\right)$$

where *V_EtOH_* is the volume of ethanol within the scaffold pores, and *V_PCL_* is the volume of PCL, which constitutes the solid fraction of the scaffold. The density of ethanol (0.790 g/mL) and bulk density of PCL (1.145 g/mL) were used to calculate the *V_EtOH_* and *V_PCL_* from the measured masses, respectively. We used 99.5% ethanol (Nacalai Tesque, Kyoto, Japan) as the displacement liquid because PCL is highly hydrophobic to ensure sufficient infiltration into the pore structure. Each measurement was recorded in triplicate.

The wettability of the scaffold surface was assessed by measuring the static contact angle with a custom-made contact angle meter. A 5 µL droplet of distilled water was gently placed onto the scaffold surface, and the contact angle was measured using the *θ*/2 method 10 s after droplet deposition to ensure stabilization of the droplet profile. Three independent samples were analyzed for each condition, and five points were averaged for each sample.

### 2.4. Fabricating and Characterizing Composite Micro/Nanofiber Scaffolds

The microfibers and submicron-fibers produced under the optimized conditions were simultaneously produced using a two-syringe configuration. The conditions that yielded the highest submicron-fiber content (polymer concentration of 9.5 wt.% and nozzle diameter of 0.55 mm) were used for producing nanofiber-dominant scaffolds, whereas the conditions that yielded the highest microfiber content (polymer concentration of 12 wt.% and nozzle diameter of 0.90 mm) were used for producing microfiber-dominant scaffolds according to the results of the single-mode fiber fabrication experiments. We used these two sets of parameters to simultaneously fabricate the composite micro/nanofibers to form fibrous scaffolds that integrated both sizes of fibers. The morphology of the composite micro/nanofibers was examined using SEM, as described in Section 2.4.

The collected fiber sheets were cut into rectangular pieces (15 × 15 mm) for the subsequent cell culture experiments. The scaffolds were sterilized via immersion in 90% ethanol for 10 min, followed by 70% ethanol for another 10 min, and then air-dried for 6 h on a clean bench prior to cell seeding. A previous study reported that ethanol-based sterilization does not adversely affect the functionality or cytocompatibility of PCL fibrous scaffolds, supporting the suitability of this method for preparing the materials for cell culture use [26]. To compare the composite scaffold with single-mode structures and to evaluate how fiber diameter distribution influences cellular responses, additional cell culture experiments were also performed using two reference scaffolds: a nanofiber-dominant scaffold, fabricated using a polymer concentration of 9.5 wt% and a nozzle diameter of 0.55 mm, and a microfiber-dominant scaffold, fabricated using a polymer concentration of 12 wt% and a nozzle diameter of 0.90 mm. These scaffolds were prepared following the same fabrication and sterilization procedures described above.

### 2.5. Cell Culture and Biological Evaluation

#### 2.5.1. Cell Culture Conditions

C2C12 murine myoblasts (RIKEN BRC Cell Bank, Tsukuba, Japan) were thawed from cryopreserved stocks and expanded in a growth medium until the required cell number was obtained for the experiments. The cells were suspended at 8.0 × 10^5^ cells/mL and seeded onto rectangular composite fiber scaffolds (15 × 15 mm) cut from the fabricated fibrous sheets.

The cells were cultured for the first two days (days 0–2) in a growth medium composed of high-glucose Dulbecco’s Modified Eagle’s Medium (DMEM; Gibco, Thermo Fisher Scientific, Waltham, MA, USA) supplemented with 10% fetal bovine serum and 1% antibiotic-antimycotic solution (Nacalai-Tesque, Kyoto, Japan). The medium was replaced from days 2 to 7 with differentiation medium consisting of high-glucose DMEM supplemented with 7% horse serum (Gibco, Thermo Fisher Scientific, Waltham, MA, USA) and 1% antibiotic–antimycotic to promote myogenic differentiation. The total culture period after cell seeding onto the rectangular scaffolds was 7 days.

#### 2.5.2. Cell Viability and Morphology

Cell viability was assessed using a live/dead staining protocol modified from a previously described method [27,28]. The cultured scaffolds were incubated in 5 mL of DMEM containing 1 μg/mL calcein-AM (Dojindo, Kumamoto, Japan) and 2 μg/mL propidium iodide (Dojindo, Kumamoto, Japan) at 37 °C for 30 min. Following the cell-staining, fluorescent images of the cells were acquired using a confocal laser scanning microscope (FV10i-DOC, Olympus, Tokyo, Japan). Viable cells emitted green fluorescence due to their intracellular calcein content, whereas dead cells were stained red with propidium iodide. Cell viability and adhesion in the inner region of the scaffold were evaluated by analyzing the fluorescent images using ImageJ software (National Institutes of Health, Bethesda, MD, USA). The images were converted to binary form, and the total area of the calcein-AM-positive regions was quantified to estimate the distribution of the viable cells within the scaffold along the thickness direction.

After the culture experiments, the medium was replaced with serum-free high-glucose DMEM supplemented with 1% antibiotic–antimycotic (Nacalai-Tesque, Kyoto, Japan) and incubated for 30 min to stabilize the cells. The cell-seeded scaffolds were fixed with 4% paraformaldehyde for 10 min and permeabilized with 0.1% Triton X-100 in PBS for 5 min. The F-actin filaments were visualized by incubating the cells with 0.7% rhodamine–phalloidin (PHDR1, Cytoskeleton Inc., Denver, CO, USA) for 30 min at 37 °C. The samples were stained with actin and then treated with a 300 nM DAPI solution (Dojindo, Kumamoto, Japan) for 5 min to stain the nuclei. The DAPI solution was then removed, and the samples were rinsed three times with PBS for fluorescence microscopy. Fluorescent images of the cytoskeletal organization and nuclei were obtained using a confocal laser scanning microscope (FV10i-DOC; Olympus, Tokyo, Japan).

The ultrastructural observations were conducted by fixing the cultured samples in 4% paraformaldehyde at room temperature for 30 min to ensure complete fixation. The samples were dehydrated using a graded ethanol series to avoid the structural collapse caused by rapid dehydration. The samples were then observed under a scanning electron microscope following the imaging procedure described in Section 2.3.

## 3. Results

### 3.1. Diameter Distribution, Porosity, and Wettability of Single-Mode Micro- and Nanofibers

**Figure 2:**
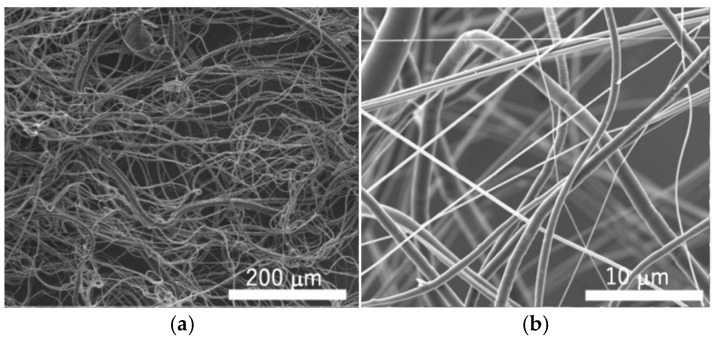
Representative scanning electron microscopy (SEM) images of fibers fabricated using centrifugal spinning device (polymer concentration: 9.5 wt.%, nozzle diameter: 0.55 mm). (**a**) Overall morphology of fibrous network; (**b**) independently acquired magnified view of the same sample, showing details of fiber structure.

Representative SEM images of single-mode fibers fabricated under various spinning conditions are shown in Figure 2. The fibers were not aligned in a specific direction but randomly extended in multiple orientations, forming an irregular 3D network rather than a planar structure. This random and spatially entangled configuration indicated that the centrifugal forces generated during the fabrication process led to fibers being deposited in various directions, resulting in a porous, volumetric structure.

**Figure 3:**
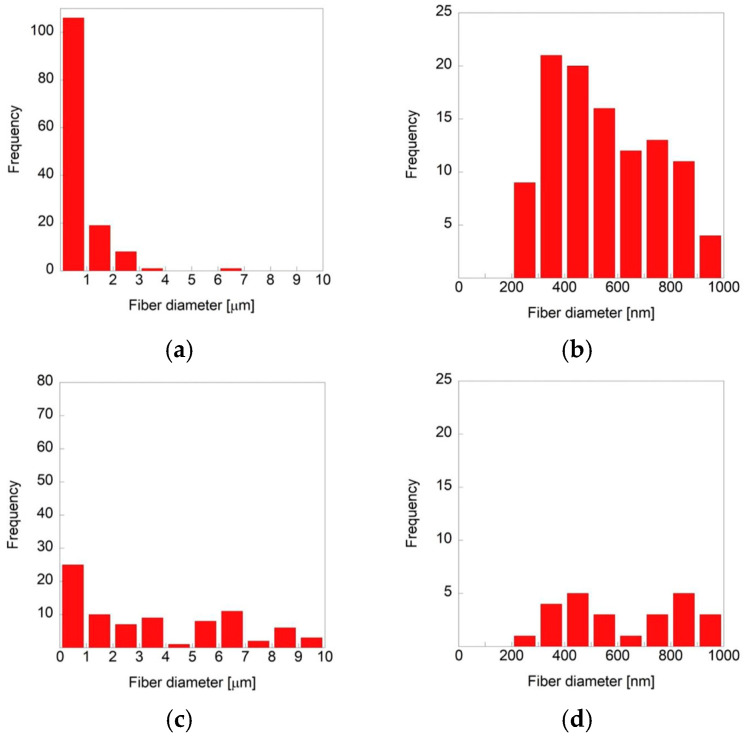
Distribution of fiber diameters produced with (**a**,**b**) 9.5 wt.% polymer solution and 0.55 mm nozzle diameter and (**c**,**d**) 12 wt.% polymer solution and 0.90 mm nozzle diameter. (**a**,**c**) Microfiber and (**b**,**d**) submicron-fiber distributions.

Microfibers and submicron-fibers were simultaneously produced under all spinning conditions, and their distribution was mixed in the fabricated fiber sheets. The relative ratio of micro- to submicron-fibers varied with the nozzle diameter and polymer concentration (Table 1). The submicron-fiber content was highest in the samples prepared with a polymer concentration of 9.5 wt.% and a nozzle diameter of 0.55 mm, while it was lowest in the sample with 12 wt.% concentration and a 0.90 mm nozzle diameter (Figure 3). The proportion of submicron-fibers decreased as the nozzle diameter increased for all polymer concentrations; higher polymer concentrations resulted in a lower submicron-fiber content for all nozzle diameters. The diameters of all fibers were less than 10 µm when the polymer concentration in the solution was 9.5 wt.% and the nozzle was 0.55 mm, and submicron-fibers accounted for 78.5% of the total fiber content (Figure 3a). The smallest fiber diameter observed under this condition was 208 nm (Figure 3b). In contrast, submicron-fibers (<1 µm) constituted 29.8% of the total fibers when a 12 wt.% solution with a 0.90 mm nozzle was used, and the smallest diameter measured was 294 nm (Figure 3c,d). These quantitative results were consistent with the SEM observations, confirming that finer fibers were predominantly generated with lower solution concentrations and smaller nozzle diameters.

The porosities of the fibrous sheets under the different experimental conditions are summarized in Table 2. The porosities exceeded 90% for all samples. The polymer concentration and nozzle diameter minimally affected the porosity under our experimental conditions.

**Figure 4:**
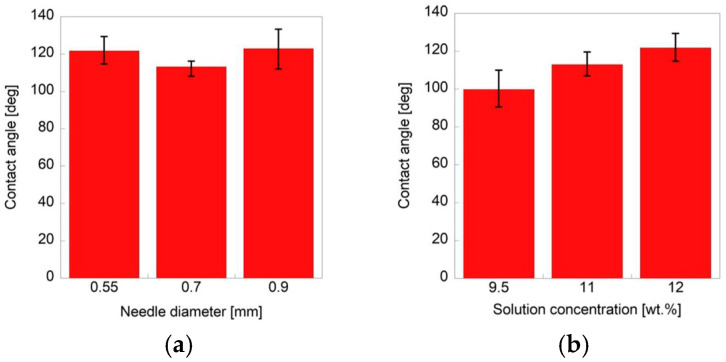
Effect of (**a**) nozzle diameter and (**b**) polymer concentration on contact angles of fiber sheets fabricated under different experimental conditions. Mean ± SD; *n* = 5.

The contact angle measurements are provided in Figure 4. We investigated the effect of the nozzle diameter on the contact angle by fixing the polymer concentration at 12 wt.%. The fiber sheets were fabricated using nozzles with diameters of 0.55, 0.70, and 0.90 mm. We also evaluated the effect of the polymer concentration on the contact angle by fixing the nozzle diameter at 0.55 mm and using solutions with 9.5, 11, and 12 wt.% polymer. The nozzle diameter did not affect the contact angle of fibers fabricated at the same concentration with different nozzle diameters. In contrast, the contact angle tended to increase with the polymer concentration for the fibers fabricated using the same nozzle diameter, indicating that the surface of the fiber sheets as more hydrophobic at higher polymer concentrations. The contact angles were larger than 90° for all samples, demonstrating that the fiber sheet surfaces were generally hydrophobic.

### 3.2. Diameter Distribution, Porosity, and Wettability of Composite Micro/Nanofiber Scaffolds

**Figure 5:**
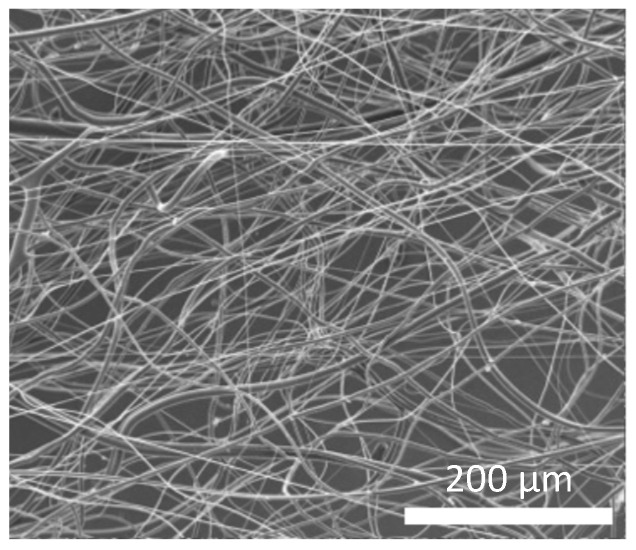
Representative SEM image of composite micro/nanofiber scaffold. Scale bar: 200 µm.

Representative SEM images of the composite micro/nanofiber scaffolds are shown in Figure 5. The fibers were randomly oriented and formed an interwoven 3D network similar to that observed in the single-mode scaffolds.

**Figure 6:**
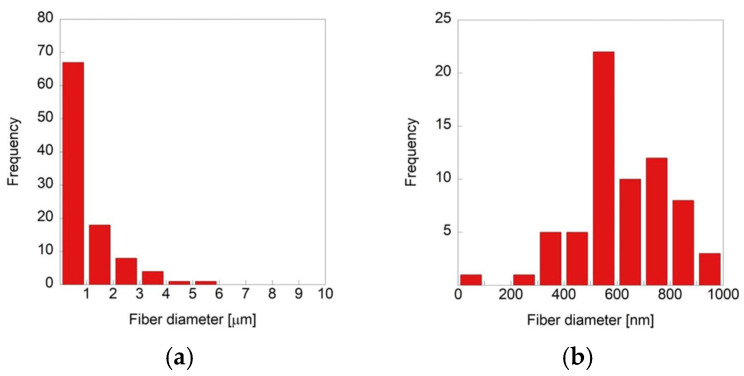
Distribution of (**a**) microfibers and (**b**) submicron-fibers in composite micro/nanofiber scaffold.

Submicron-fibers accounted for 67.0% of the total fiber population (Figure 6a). The smallest measured fiber was 97.8 nm in diameter (Figure 6b). The proportion of submicron-fibers was lower than that obtained under the nanofiber-dominant condition (polymer concentration of 9.5 wt.% and nozzle diameter of 0.55 mm) but higher than that obtained under the microfiber-dominant condition (polymer concentration of 12 wt.% and nozzle diameter of 0.90 mm). These results confirmed that the dual spinning process successfully produced a hybrid fibrous scaffold containing micro- and nanofibers in balanced proportions.

The composite fiber scaffolds were highly porous, at 95.6%, which was comparable to that of the single-mode fiber samples, the porosities of which all exceeded 90%. The contact angle of the composite scaffolds was 100.5°, which was also between the values obtained for the single-mode nanofiber-dominant and microfiber-dominant sheets. This finding suggested that the hydrophobicity of the composite scaffolds was moderate, in aligned with their mixed fiber morphology.

### 3.3. Cell Viability and Morphology on Composite Micro/Nanofiber Scaffolds

**Figure 7:**
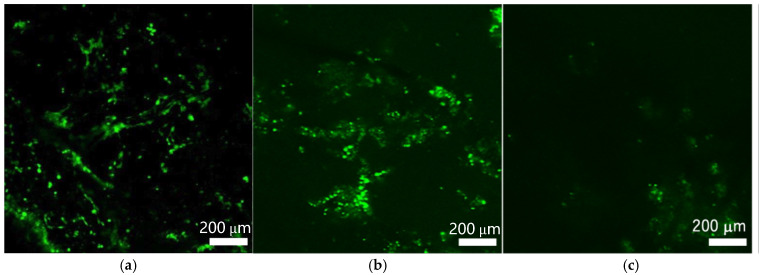
Representative fluorescent microscopic images of C2C12 cells grown on (**a**) micro/nano fiber, (**b**) nanofiber-dominant, and (**c**) microfiber-dominant scaffolds. Microscopic images were captured at a depth of 25 µm from scaffold surface.

Representative live/dead fluorescence images of C2C12 cells cultured on the composite micro/nanofiber, nanofiber-dominant, and microfiber-dominant scaffolds at a depth of 25 µm from the surface are shown in Figure 7. Viable cells stained with calcein-AM (green) and dead cells stained with propidium iodide (red) were observed in both the composite and nanofiber-dominant scaffolds, whereas only a small number of cells were detected on the microfiber-dominant scaffold.

**Figure 8:**
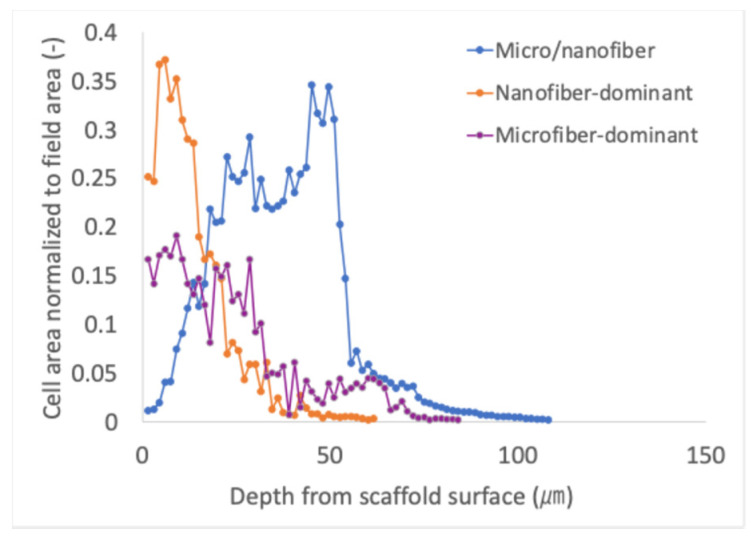
Depth-dependent distribution of viable cells within scaffold based on area stained with calcein-AM.

In the composite micro/nanofiber scaffold, most cells localized within a depth of approximately 110 µm from the scaffold surface, whereas in the nanofiber-dominant scaffold, a high density of cells was observed only near the surface region, with significantly fewer cells detected at deeper positions (Figure 8). Only a few dead cells were present, indicating that the scaffold maintained high cell viability during culture. Although cell density was relatively low near the surface, a higher density of viable cells was detected at depths between 20 and 60 µm in the composite micro/nanofiber scaffold. Considering that the scaffold thickness was approximately 250 µm, these results suggest that cells infiltrated and adhered to nearly half of the scaffold thickness from the top surface when cells were cultured on the composite scaffold. In contrast, cells cultured on the nanofiber-dominant scaffold were mainly distributed within the region between 0 and 30 µm from the surface. For the microfiber-dominant scaffold, only a limited number of cells were detected across all depths.

**Figure 9:**
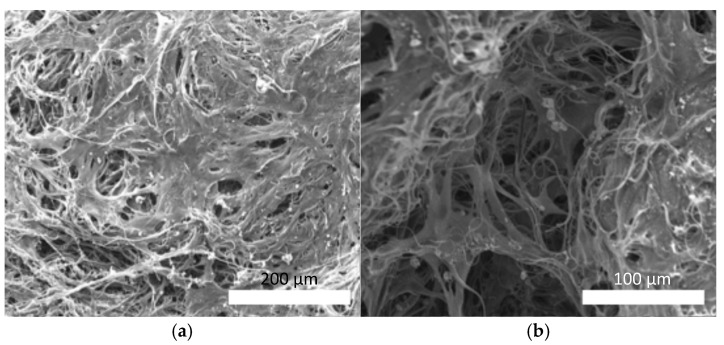
Representative SEM images of C2C12 cells cultured on composite micro/nanofiber scaffold. (**a**) Cells adhered and proliferated on scaffold surface, covering and filling interfiber spaces (scale bar: 200 µm). (**b**) Cells adhered along fibers within inner scaffold region (scale bar: 100 µm).

The SEM observations (Figure 9) revealed differences in the cell morphology between the surface and interior regions of the scaffolds. The cells adhered and proliferated to fill the interfiber spaces on the scaffold surface (Figure 9a), forming a continuous tissue-like layer that partially covered the fiber network. In contrast, the cells tightly adhered along the fibers within the inner regions of the scaffold, extending the morphology in the direction of the local fiber orientation (Figure 9b). The cells were elongated, suggesting that the fiber alignment guided the cell spreading along the fibrous structure.

**Figure 10:**
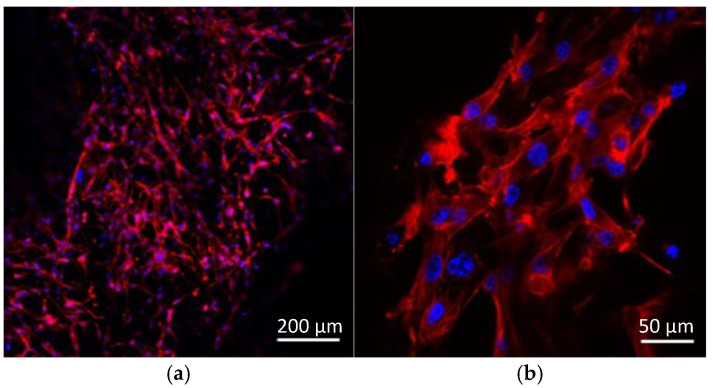
Representative confocal fluorescence images of C2C12 cells cultured on composite micro/nanofiber scaffold. (**a**) Actin filaments (rhodamine–phalloidin, red) and nuclei (DAPI, blue) showing well-developed cytoskeletal structures aligned along underlying fibers (scale bar: 200 µm). (**b**) Magnified view showing multiple nuclei positioned in close proximity within single cells, indicating multinucleated myotube-like morphology (scale bar: 50 µm).

Confocal fluorescence images of actin filaments stained with rhodamine–phalloidin and nuclei stained with DAPI are shown in Figure 10. The cytoskeletal structures were well-developed, and actin filaments were aligned along the underlying fibers, demonstrating that the cells extended and organized their cytoskeleton along the fibrous structure (Figure 10a). In addition, multiple nuclei were observed in close proximity, indicating the presence of multinucleated cells with a myotube-like morphology on the composite scaffolds (Figure 10b).

## 4. Discussion

In this study, the fiber-spinning device using two pairs of counter-facing syringes was developed to simultaneously eject and form fibers under multiple processing conditions. This system was developed based on the conventional forcespinning approach [23,24,25] to fabricate composite fibrous scaffolds with controlled fiber diameter polydispersity, thereby integrating the mechanical stability and structural porosity associated with microfibers with the cell-adhesive characteristics typically attributed to nanofibers. These hybrid scaffolds could provide structural integrity and enhanced biological functionality in tissue engineering applications.

Nanofibers are mainly produced by electrospinning, which yields fibers with highly uniform diameters [29,30]. Although several scale-up approaches such as multi-nozzle systems [19] and hierarchical micro/nano multilayered scaffolds [22] have been proposed to improve productivity and incorporate multiple fiber sizes, electrospinning cannot simultaneously generate fibers with different diameters that are homogeneously integrated within a single structure. Although inferior to electrospinning in terms of diameter uniformity, our proposed device enables the fabrication of fibrous scaffolds with controlled fiber diameter polydispersity spanning from microscale to nanoscale fibers, which represents a promising approach for developing composite fibrous scaffolds for regenerative medicine.

The fibers were formed in this study by ejecting a PCL solution through needle-type nozzles using centrifugal force. The elongated jet solidified as the solvent, 2,2,2-trifluoroethanol, evaporated during flight and was collected on a cylindrical trap plate. The diameter distribution of fibers ranged from nanoscale to microscale. Maximum ratio of submicron-fibers (<1 µm) was around 80%, which is a lower value as compared to that of fibers fabricated by electrospinning. Although this broad diameter distribution is the limitation of centrifugal spinning approach, it is expected that the diameter distribution of fibers by our centrifugal spinning method could be more homogeneous by maintaining the fabrication conditions such as solvent concentration, rotational speed, needle size, and ambient temperature [31].

The average fiber diameter decreased with smaller nozzle diameters, which is geometrically reasonable, and increased with higher polymer concentrations. The viscosity of the polymer solution, which rises markedly with concentration, is known to be a key parameter governing fiber diameter in centrifugal spinning [31]. As viscosity increases, the viscous resistance reduces the relative velocity between the solution and the inner nozzle wall, effectively decreasing the flow rate during ejection. This reduced flow rate suppresses jet elongation and thinning, resulting in thicker fibers at higher polymer concentrations.

The porosities of the fabricated fiber sheets all exceeded 90%; the highest porosity was 95.6% for the composite scaffolds. These values are comparable to or higher than those reported for conventional fibrous cell culture scaffolds [32,33,34], indicating that the developed structures are appropriate for cell culture. The porosities of fabricated fiber scaffolds in this study also showed similar values to those fabricated by novel electrospinning approach with controlling fiber collector positions [21]. High porosity facilitates nutrient exchange and cellular infiltration while maintaining scaffold integrity.

The contact angle analysis revealed that the wettability of the fiber sheets decreased (i.e., the contact angle increased) as the polymer concentration increased, corresponding to a higher proportion of microfibers in the scaffold. Because nanofibers possess a much larger surface area per unit volume, their increased presence leads to a greater exposure of the material surface. As a result, the larger surface area of nanofiber-dominant scaffold exhibited enhanced hydrophilicity, which was reflected by the smaller contact angles observed.

The composite fiber sheets fabricated using the fiber-spinning device with two pairs of counter-facing syringes contained fibers spanning from microscale to nanoscale within a single scaffold. Although only the combination of two processing parameters was tested in this study, the spinning speed, polymer concentration, atmospheric temperature, and nozzle diameter may be varied to more accurately control the distribution of the fiber diameter. Moreover, increasing the number of opposing syringe pairs could enable the modulation of the ratio of micro- to nanofibers, allowing the scaffold morphology to be tailored to specific applications.

The cell culture experiments demonstrated that all fabricated scaffolds were non-cytotoxic, as evidenced by the high viability of C2C12 myoblasts across all conditions. PCL is a biocompatible polymer for tissue engineering [18,35,36,37,38], and no residual cytotoxicity was observed because the solvent 2,2,2-trifluoroethanol likely completely evaporated during spinning. The composite micro/nanofiber scaffold supported not only cell adhesion but also deeper cellular infiltration, with cells infiltrating approximately half of scaffold thickness. This behavior indicates that the composite architecture provides an appropriate balance of pore size and fiber density, enabling cells to infiltrate into the interior while still offering sufficient anchoring points for attachment. In contrast, cell distribution on the nanofiber-dominant scaffold was confined to the superficial region. The dense nanofiber network likely reduced pore size to decrease the cell infiltration, causing most cells to remain near the surface. Conversely, only a limited number of cells were detected within the microfiber-dominant scaffold. The relatively large pore structures created by thick microfibers were considered too large to effectively retain or trap cells, allowing them to pass through without stable cell adhesion. These findings suggest that neither nanofiber-only nor microfiber-only architectures alone provide an optimal microenvironment for both cell retention and infiltration. The composite scaffold, integrating micro- and nanoscale fibers within a single structure, achieves a more favorable pore architecture that promotes both surface adhesion and three-dimensional cellular integration.

In this study, the cell suspension was dropped onto the upper surface of the scaffold. The cells were primarily distributed within the upper half of the scaffold thickness, even when using the composite micro/nanofiber scaffold. Cells could be seeded from both sides or dynamic seeding methods, such as perfusion culture [39,40,41], could be used in future studies to more homogeneously distribute the cells throughout the scaffold. Furthermore, an excessive nanofiber content could hinder the infiltration of cells into the scaffold interior because the nanofibers fabricated in this study were approximately one-tenth the size of the cells. Therefore, the micro/nanofiber ratio should be optimized for improving scaffold performance.

C2C12 myoblasts are model cells used for skeletal muscle differentiation, which we adopted to evaluate the biological performance of the fabricated fibrous scaffolds. The cells adhered, spread, and formed multinucleated structures, demonstrating that the fibrous scaffold supported myoblast maturation. These results suggest that the developed composite fiber scaffolds can be used as culture substrates for muscle, tendon, and ligament tissue engineering, in which fibrous architectures play key structural and functional roles. However, only one kind of cell was tested for evaluation of biocompatibility and cytotoxicity in this study. In the future study, cell culture tests using various kinds of cell types would be required to confirm the biocompatibility and cytotoxicity.

## 5. Conclusions

A centrifugal fiber-spinning device using two pairs of counter-facing syringes was developed to fabricate fibrous scaffolds containing fibers spanning from microscale to nanoscale within a single structure. By adjusting the nozzle diameter and polymer concentration, the fiber diameter polydispersity could be systematically controlled, enabling modulation of the proportion of submicron fibers from approximately 30% to 80%. In addition, all fabricated scaffolds exhibited high porosity exceeding 90%, indicating that the proposed approach allows simultaneous control of fiber diameter distribution and scaffold openness through well-defined processing parameters.

Cell culture experiments using C2C12 myoblasts revealed distinct differences in cell infiltration behavior depending on the scaffold architecture. On the composite micro/nanofiber scaffold, viable cells infiltrated beyond the surface region and were distributed throughout the middle layer of the scaffold. In contrast, cells cultured on the nanofiber-dominant scaffold were primarily confined to the surface region. For the microfiber-dominant scaffold, only a limited number of cells were detected across all regions. In all cases, few dead cells were observed, confirming the noncytotoxic nature of fabricated scaffolds. The developed composite fiber scaffold therefore represents a promising functional substrate for fibrous tissue engineering.

## Figures and Tables

**Table 1:** Ratio of submicron-fibers (diameter < 1 µm) in fiber sheets fabricated under different experimental conditions.

Polymer Concentration (%)	Nozzle Diameter (mm)	Ratio of Fibers with Diameters < 1 µm
9.5	0.55	78.5
	0.70	75.2
	0.90	58.7
11	0.55	66.9
	0.70	53.1
	0.90	32.1
12	0.55	57.0
	0.70	33.0
	0.90	29.8

**Table 2:** Porosity of fiber sheets fabricated under different experimental conditions (*n* = 5, Mean ± SD).

Polymer Concentration (%)	Nozzle Diameter (mm)	Porosity (%)
9.5	0.55	90.4 ± 1.5
	0.70	93.6 ± 0.3
	0.90	94.3 ± 0.9
11	0.55	93.2 ± 2.2
	0.70	95.4 ± 0.6
	0.90	93.6 ± 0.9
12	0.55	94.3 ± 0.2
	0.70	92.2 ± 1.9
	0.90	93.2 ± 1.2

## Data Availability

The datasets presented in this article are not readily available because they are part of an ongoing project. Access to the datasets should be directed to the corresponding author.

## Abbreviations

The following abbreviations are used in this manuscript:

ECM	Extracellular matrix
PCL	Poly(ε-caprolactone)
SEM	Scanning electron microscopy
DMEM	Dulbecco’s modified Eagle medium
